# New Trend in Toxicological Screening Using Volumetric Absorptive Microsampling (VAMS) and High-Resolution Mass Spectrometry (HR/MS) Combination

**DOI:** 10.3390/molecules28083466

**Published:** 2023-04-14

**Authors:** Pascal Houzé, Ilona Borowski, Eugénie Bito, Romain Magny, Athina Morcos, Sebastian Voicu, Bruno Mégarbane, Laurence Labat

**Affiliations:** 1 Laboratory of Toxicology, Federation of Toxicology, Lariboisière Hospital, Assistance Publique-Hôpitaux de Paris (AP-HP), 10 rue Ambroise Paré, 75010 Paris, France; 2 Chemical and Biological Health Technologies Unit (UTCBS), CNRS UMR8258-U1022, University of Paris, 4 Avenue de l’Observatoire, 75006 Paris, France; 3 INSERM UMRS-1144, University of Paris, 4 Avenue de l’Observatoire, 75006 Paris, France; 4 Department of Medical and Toxicological Critical Care, Federation of Toxicology, Lariboisière Hospital, Assistance Publique-Hôpitaux de Paris (AP-HP), 10 rue Ambroise Paré, 75010 Paris, France

**Keywords:** toxicological screening, whole blood, volumetric absorptive microsampling (VAMS), Mitra^TM^ device, high-resolution mass spectrometry, clinical application

## Abstract

In toxicology, screenings are routinely performed using chromatographic methods coupled to detection systems such as high-resolution mass spectrometry (HR/MS). The increase in specificity and sensitivity of HRMS is responsible for the development of methods for alternative samples such as Volumetric Adsorptive Micro-Sampling. Whole blood overloaded with 90 drugs was sampled with 20 µL Mitra^TM^ to optimize the pre-analytical step as well as to determine the identification limits of drugs. Elution of chemicals was carried out in a solvent mixture through agitation and sonication. After dissolution, 10 μL was injected into the chromatographic system coupled to the Orbitrap^TM^ HR/MS. Compounds were confirmed against the laboratory library. The clinical feasibility was assessed in fifteen poisoned patients using the simultaneous sampling of plasma, whole blood and Mitra^TM^. The optimized extraction procedure allowed us to confirm 87 compounds out of the 90 present in the spiked whole blood. Cannabis derivatives were not detected. For 82.2% of the investigated drugs, the identification limits were below 12.5 ng·mL^−1^, with the extraction yields ranging from 80.6 to 108.7%. Regarding the patients’ analysis, 98% of the compounds in plasma were detected in Mitra^TM^ compared to whole blood, with a satisfying concordance (R^2^ = 0.827). Our novel screening approach opens new insights into different toxicologic fields appropriate for pediatrics, forensics or to perform mass screening.

## 1. Introduction

Toxicological screening represents one of the core activities in all toxicology laboratories to detect both licit and illicit compounds in various matrices. Screenings are performed in different contexts, such as forensics, to determine the causes of death, doping, and, more commonly, clinical toxicology [1,2]. Usually performed through the analysis of plasma or urine, the aim of clinical toxicology screening is to improve the management of the patient by the clinician, especially if the management requires the use of an antidotal or purifying treatment. It may also be a useful approach when there is a need for increased monitoring when the molecules may be responsible for serious or delayed complications beyond certain plasmatic concentrations [3]. To fulfill these challenges, analytical methods of screening have been developed through different approaches. They may be based on immuno-screening methods, which are easy to implement but have limited interest regarding specificity [4]. Furthermore, the evolution of separative methods and the combination with mass spectrometry has led to significant changes in toxicological screening both for sensitivity and specificity. Historically, the first efficient screenings were based on gas chromatography hyphenated to electron impact mass spectrometry (GC/EI-MS) thanks to the facilities of coupling gas chromatography with a mass spectrometer. These methods are well suited to detect different classes of compounds, such as barbiturates, amphetamines, cannabinoids, new psychoactive substances (NPS), and doping substances in various biological matrices [5,6,7,8]. However, their use is limited to thermally stable and hydrophobic compounds [9]. These limitations have been overcome using derivatization techniques, which allows the chemical modification of hydrophilic functions, improving both the thermal stability of molecules and the sensitivity. Their routine application has enabled the generalization of screening using GC/EI-MS in various biological matrices to a wide range of compounds [10]. Furthermore, the inter-laboratory reproducibility, thanks to the use of electron impact as an ion source, has been conducted to consider GC-EI-MS as a gold standard approach for toxicological screening.

Liquid chromatography (LC) has significantly improved the possibilities of toxicological screening. The diversity of molecules that can be separated using LC is wide, regardless of their nature, size, and physicochemical properties. The reduction in particle size of the stationary phase has resulted in enhanced resolution and chromatographic separations, comparable in some cases to those obtained with gas chromatography. Finally, the ability to couple liquid chromatography to increasingly sensitive and specific detection systems is a key advantage in toxicological screening. Initially based on diode array detection [11,12,13], today, toxicological screening is routinely performed using liquid chromatography hyphenated to a simple quadrupole (LC-MS) or, more commonly, to a tandem mass spectrometer (LC-MS/MS) [14,15,16]. The development of high-resolution mass spectrometry (HR/MS) based on Orbitrap™ [17,18] or Time of Flight (TOF) [17,19] technologies is one of the latest significant advances in the field of toxicological screening, either through a targeted or non-targeted approach [20,21]. High-resolution mass spectrometry offers the possibility of identifying compounds with a precision of one-millionth of an atomic mass. Nevertheless, it must be noted that these methods have significantly increased the specificity of detection, sometimes at the expense of sensitivity. Indeed, a high-resolution mass spectrometer operating under full scan mode for a screening procedure may reduce the sensitivity compared to the classically selected reaction monitoring approach used in conventional LC-MS/MS screening [17]. However, the broad number of compounds identified using this approach may compensate for the loss of sensitivity.

In addition to these analytical advances, the reduction in sample volume and the use of alternative matrices may be regarded as recent developments in the field of toxicological analysis. The use of alternative matrices, such as dried blood spots (DBS), has been reported for several years in different fields of toxicology including forensic [22], doping [22,23,24] and drug detection [22,25,26]. These systems have several advantages, including a low sampling volume and the fact that they are minimally invasive with facilitated transport and storage [25,26]. However, their use is generally limited to qualitative applications due to the inaccuracy in sampling volume [25,26]. Indeed, the hematocrit value has been identified as the main cause of variability in the volume collected on DBS [27,28]. The development of Volumetric Adsorptive Microsampling (VAMS) systems combined the advantages of DBS with the possibility of precise sampling regarding the collected volume, independently of the hematocrit value [29]. Among the different available VAMS systems, Mitra^TM^ devices are one of the most frequently used [28]. Using such devices, numerous applications in the field of therapeutic drug monitoring, especially for immune suppressants [30], antiepileptic drugs [31], or antidepressants [32] have been previously reported. Regarding the toxicological field, a VAMS combined with LC-MS/MS was developed to detect and quantify illicit drugs, such as cocaine [33], natural and synthetic cannabinoids [26] or cathinones in different biological matrices, such as blood, plasma, urine and saliva [34]. To our knowledge, only one publication reports toxicological screening on whole blood sampled using Mitra^TM^ devices combined with LC-MS/MS during roadside checks [35]. In our study, dealing with a clinical context, we aimed to develop a method for toxicological screening using a VAMS system, namely Mitra^TM^ one. Our method combines the advantages of microsampling, by working on small volume samples, easy to collect and easy to preserve, with the high sensitivity and specificity of LC-HR/MS. Among these advantages, the mass precision brought using high resolution allows us to identify, with a high degree of certainty, many compounds with various structures and physicochemical properties. To our knowledge, such a combination of methods has never been developed in clinical toxicology. The objective of this work was, thus, to demonstrate that a reliable toxicological screening to detect both licit and illicit drugs may be performed through 20 µL of whole blood sampled on a Mitra^TM^ device using an analytical procedure based on LC-HR/MS detection. After performing optimization steps, our new screening method was applied to patients hospitalized for intoxication to attest to its feasibility in a clinical setting.

## 2. Results

We developed a three-step process to optimize and validate our new approach as useful for a reliable toxicological screening (steps 1 and 2) and to assess its clinical application in real life (step 3).

### 2.1. Step 1: Optimization of the Extraction Conditions from Mitra^TM^ Devices

**Figure 1:**
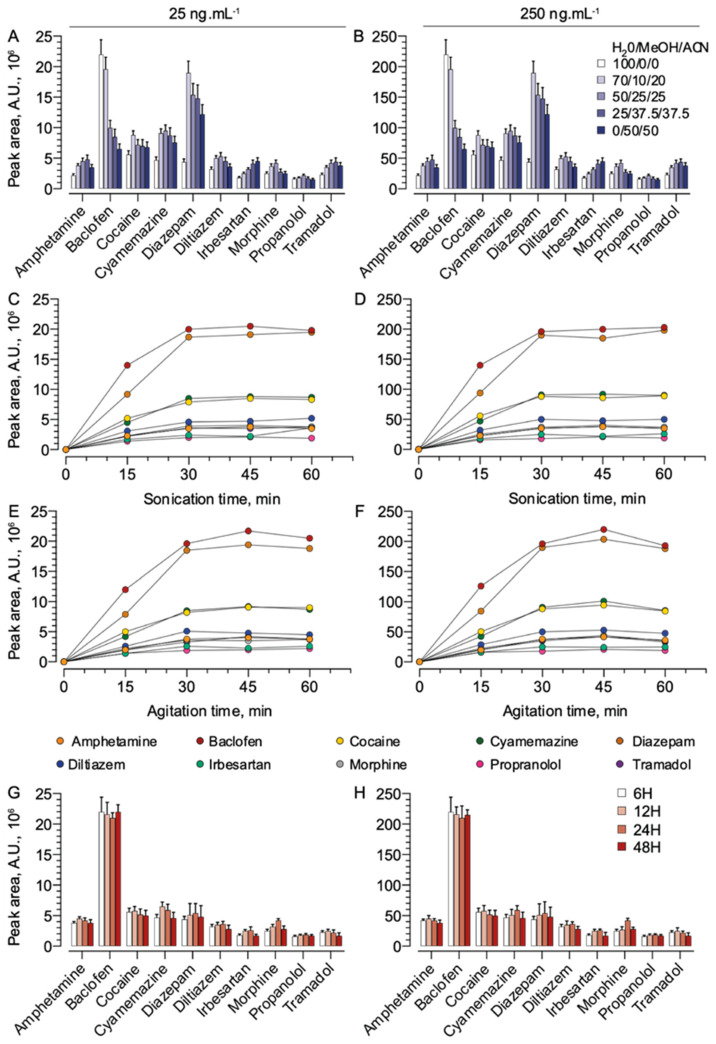
Effects of the extraction mixture’s composition (**A**,**B**), sonication duration (**C**,**D**), agitation duration (**E**,**F**), and the time elapsed between filling the Mitra^TM^ devices and the extraction (**G**,**H**) on the response intensity of 10 selected molecules at 25 and 250 ng·mL^−1^. Results are expressed as the mean ± standard deviations of three independent replicates. A.U.: Arbitrary Unit.

The extraction conditions of ten selected molecules were optimized at two concentrations (25 and 250 ng·mL^−1^) according to three parameters: composition of the extraction mixture (Figure 1A,B); sonication times (Figure 1C,D); agitation times (Figure 1E,F) and the delay between the filling of the Mitra^TM^ device and the extraction (Figure 1G,H). For the two investigated concentrations, as displayed in Figure 1A,B, an optimal mixture of 75% water with 1% formic acid combined with 10% methanol and 15% acetonitrile was established as the most efficient to extract all the compounds included in this study, regardless of their structure and solubility. As an example, for baclofen, which is a hydrophilic molecule, extraction using only water with 1% FA led to the highest extraction yield while more lipophilic compounds were not efficiently extracted (Figure 1A,B). Indeed, for molecules displaying a lipophilic character, as is the case for irbesartan and cyamemazine, the extractions were favored by increasing the organic fraction in the mixture (Figure 1A,B). Finally, the use of a mixture containing only organic solvents (methanol/acetonitrile, 50/50%, *v*/*v*) resulted in a decrease in signal intensity for most of the investigated molecules, highlighting the need for water within the extraction mixture.

Figure 1C–F illustrates the impact of the sonication and agitation times on the intensity of the responses for 10 of the investigated molecules. The most intense areas were obtained with 30 min of sonication followed by 30 min of agitation for all compounds. Furthermore, an additional agitation and/or extraction time did not significantly increase the response intensity.

In a final optimization step, we investigated the influence of the delay between the filling of the Mitra^TM^ device and the extraction time. As shown in Figure 1G,H, the area of the investigated drugs remained consistent for delays between 6 and 72 h.

### 2.2. Step 2: Determination of Identification Limits and Extraction Yields for 90 Compounds Sampled Using Mitra^TM^ Devices

Following the optimization of the extraction steps, we then engaged in the determination of the identification limits and extraction yields of 90 compounds. The choice of the 90 molecules, including both legal and illegal drugs, selected to establish their identification limits was based on the selection of chemicals with different physicochemical properties, and those belonging to various pharmacological classes. Regarding our 3 years of experience in plasma toxicological screening using LC-HR/MS in patients hospitalized in the toxicological critical care unit, these compounds are commonly found in more than 50% of hospitalized patients.

**Figure 2:**
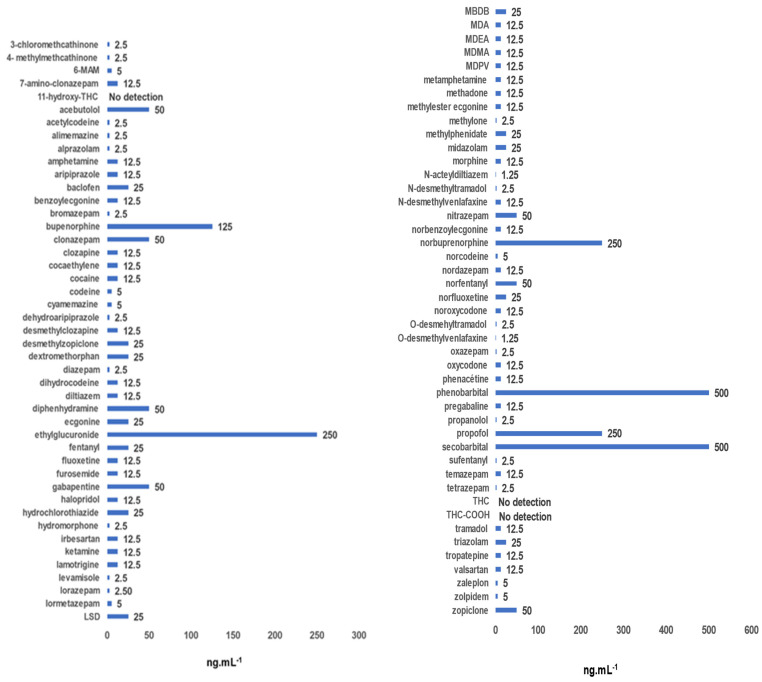
Limits of identification of 90 compounds spiked in working solutions at different concentrations from 250 to 1.25 ng·mL^−1^. The values of the identification limits are specified for each compound at the top of each bar. 6-MAM: 6-monoacetylmorphine; 11-hydroxy-THC: 11-hydroxy-Δ9-tetrahydrocannabinol; LSD: lysergic acid diethylamide; MBDB: N-methyl-1-(1,3-benzodioxol-5-yl)-2-aminobutane; MDA: 3,4-méthylènedioxyamphétamine; MDEA: 3,4-méthylènedioxy-N-éthylamphétamine; MDMA: 3,4-methylenedioxy-N-methylamphetamine; MDPV: Methylenedioxypyrovalerone; THC: tetrahydrocannabinol; THC-COOH: 11-carboxy-tetrahydrocannabinol.

Limits of identification (LOI) can be defined as the lowest analyte concentration that can be confirmed according to the parameters described in the Data Analysis section. Using the sampling based on the Mitra^TM^ device and our analytical procedure, three compounds out of 90, i.e., 3.3% of the tested substances, were not detected. Furthermore, six molecules, including barbiturates, buprenorphine, ethyl glucuronide and propofol were only detected and confirmed at concentrations above 125 ng·mL^−1^. For seven other compounds, including acebutolol, clonazepam or zopiclone, the identification limits were determined to be 50 ng·mL^−1^, which may be considered satisfactory. For the remaining 74 compounds, i.e., 82.2% of the investigated compounds, the identification limits were all very satisfactory, below 12.5 ng·mL^−1^, allowing their detection and identification according to the above-described criteria (Figure 2).

For the 74 compounds detected, the extraction yields were determined using the ratio of the areas of the chromatographic peaks between the Mitra^TM^ devices and the whole blood. The extraction yields ranged from 80.6% for diazepam to 108.7% for cyamemazine.

### 2.3. Step 3: Clinical Application

**Figure 3:**
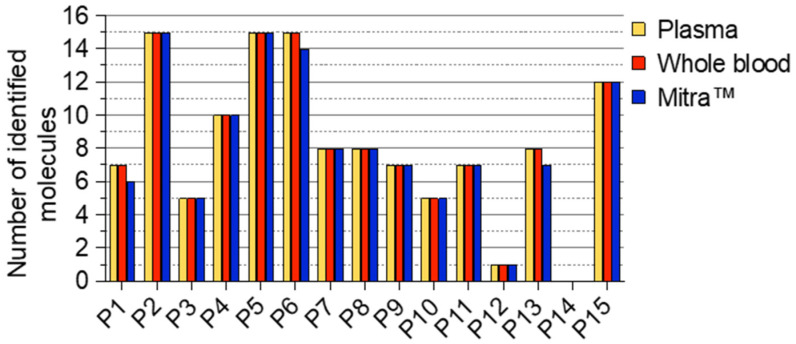
Comparison of the number of molecules identified in the different samples for the 15 patients hospitalized in the toxicological critical care unit.

Following the optimization of the extraction conditions to achieve good sensitivity and good yield for compound detection, we then assessed the feasibility of our newly developed screening method in real life through its application to 15 patients hospitalized in the toxicological critical care unit. Figure 3 shows the number of molecules detected in the three investigated samples: plasma, whole blood and Mitra^TM^. The same number of compounds, ranging from 0 to 15, were detected and confirmed in the plasma and whole blood of all patients. The comparison between the whole blood and the Mitra^TM^ devices showed an entire concordance for 12 to 15 patients. Nevertheless, for three patients (P1, P6, and P13), two molecules were not detected using the Mitra^TM^ devices as the sampling method; these correspond to diazepam (P1 and P13) and ethyl glucuronide (P6).

**Figure 4:**
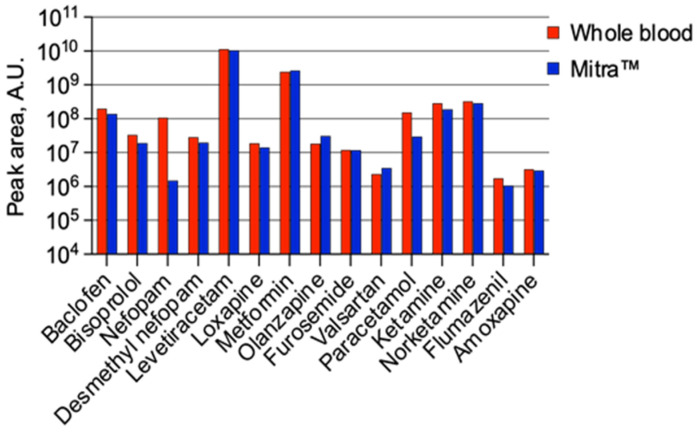
Details of the molecules identified in patient P2 in the whole blood samples and Mitra^TM^ device. A.U.: Arbitrary Unit.

**Figure 5:**
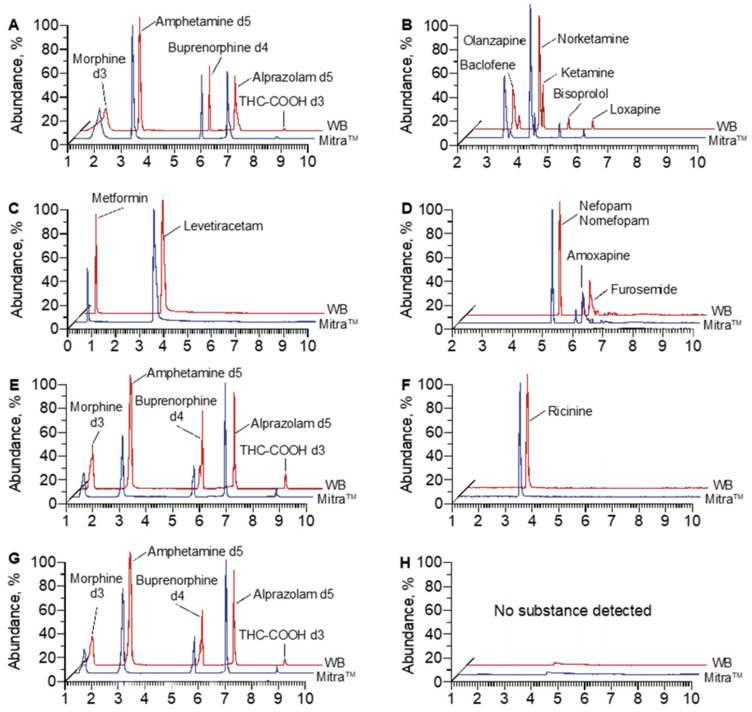
Examples of the chromatograms obtained for patient P2 ((**A**) Internal Standards, (**B**–**D**) identified molecules), patient P12 ((**E**) Internal Standards, (**F**) identified molecule) and patient P14 (**G**) Internal Standards, (**H**) identified molecule) after extraction using Mitra^TM^ devices.

Figure 4 and Figure 5 provide detailed information on the identified compounds in the whole blood and Mitra^TM^ device for patient P2, including the drugs used in intensive care during reanimation (nefopam and desmethyl nefopam, ketamine and norketamine, furosemide), the usual treatment of the patient (baclofen, levetiracetam, bisoprolol, metformin), as well as loxapine and olanzapine, molecules ingested by the patient in a suicidal attempt. The screening procedure also made it possible to detect amoxapine, an active metabolite of loxapine.

Finally, 122 compounds, including parent molecules and metabolites, were detected in the whole blood samples compared to 119 from the Mitra^TM^ samples. This corresponds to a detection efficiency of 98%. Among the identified compounds, benzodiazepines (alprazolam, bromazepam, diazepam and metabolites) and related compounds (zolpidem, zopiclone) were detected in 50% of the investigated patients; antidepressant drugs (amitriptyline, paroxetine, loxapine, amoxapine, citalopram) in 40%; cardiotropic drugs (bisoprolol, propranolol, amiodarone) in 32%; phenothiazines (alimemazine, cyamemazine) in 15%; and neuroleptics (haloperidol, aripiprazole) in 10%. In less than 1% of the patients, a hypoglycemic drug (metformin), antidotes (N-acetylcysteine, flumazenil) and opiates (morphine, codeine) were also detected. For one patient (P12), only one molecule was identified in all samples, ricinine, following the ingestion of several nuts of the castor plant with suicidal intent (Figure 3 and Figure 5). No compounds were detected in any samples of patient P14 (Figure 3 and Figure 5). Among the illicit drugs, only cocaine and its metabolites (benzoylecgonine, ecgonine methyl ester, ecgonine, nor benzoylecgonine coca ethylene) were identified in 25% of patients. No other illicit drugs were identified in the investigated samples.

**Figure 6:**
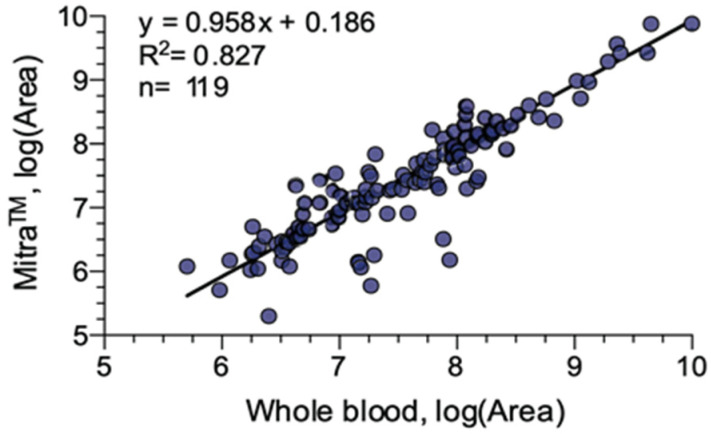
Correlation between the chromatographic peak areas of 119 molecules identified in whole blood and Mitra^TM^ devices.

**Figure 7:**
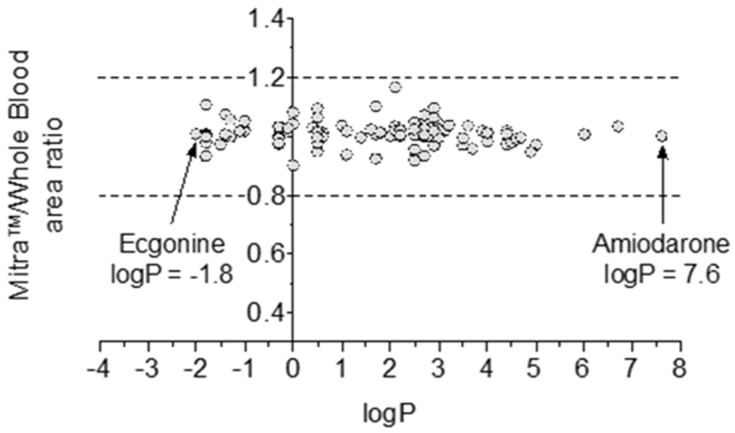
Influence of the log *p* value on the extraction yields of 119 identified molecules from the Mitra^TM^ devices.

The correlation between the areas of the chromatographic peaks obtained from the Mitra^TM^ devices compared to those obtained from whole blood is satisfactory, with an R^2^ value of 0.82 for the 119 compounds identified (Figure 6). However, for the nine molecules displayed in Figure 6, the correlation appears to be weaker; and these compounds may be regarded as outliers. This corresponds to different compounds, identified in two cases as etomidate, two cases as nefopam, and in one case as either midazolam, hydroxyl-midazolam, cocaine, cocaethylene or propranolol. All of these compounds have log *p* values ranging from 2.3 for cocaine to 3 for nefopam and propranolol. Log *p* values denote the lipophilic/hydrophilic character of a molecule, which can strongly influence the molecule’s extraction from matrices. To investigate the impact of hydrophobicity on the extraction of the 119 confirmed molecules, we have reported the extraction yields for each molecule according to their log *p* value (Figure 7). For all of the molecules, the extraction yields were within the accepted values of 80 to 120%, both for very hydrophilic molecules such as ecgonine (log *p* = −1.8) or very lipophilic such as amiodarone (log *p* = 7.6).

## 3. Discussion

In toxicological analysis, a major evolution has been made through the combination of increasingly sensitive and specific methods and the use of microsamples [36]. Among the microsampling systems, DBS have been used in different fields of biology since the 1960s [37]. In toxicology, DBS were successfully applied to detect illicit drugs including natural and synthetic cannabinoids [26,38], opiates [39], 3,4-methylenedioxymethamphetamine (MDMA) [40], γ-hydroxybutyric acid (GHB) [41] and cocaine [42]. Despite the many advantages of DBS, their main limit remains in the uncertainty of the sampling volume due to hematocrit value variations. To overcome this drawback, in a study involving healthy volunteers, the authors did not consider the hematocrit value as it was within the physiological norms for all participants [42]. On the other hand, a study on the quantification of MDMA does not report this issue [40], while a 2010 paper lists all of the limits of using DBS for quantitative studies, especially the uncertain volume of the sample [43]. The development of a new microsampling system, VAMS, which is not, or less, affected by hematocrit values, has allowed for the development of quantitative methods. These methods have been rapidly applied in different fields, especially in therapeutic drug monitoring [30,31,32]. In toxicology, several publications report the quantification of various drugs from the VAMS system, focusing on the quantification of one drug and its metabolites such as cocaine [42], or on a family of compounds such as the cathinones [35]. In 2021, a publication reported on a toxicological screening using the VAMS system but was limited to the identification and quantification of 18 compounds [34]. The use of the LC-MS/MS approach in these different studies may explain the limited number of identified molecules. Furthermore, it is well known that LC-MS/MS does not allow for such extensive detection as when LC-HR/MS is used. In this work, the use of LC-HR/MS allowed for the detection of over 1500 molecules, compared to a reference library, upon the analysis of a 20 µL extract of whole blood collected using the Mitra^TM^ device. This study is the first to combine HR/MS and VAMS microsampling for extended toxicological screening purposes. Indeed, only Joye et al. [44] have used Orbitrap™ High Resolution for extended screening from whole blood DBS in clinical and forensic toxicology.

In the framework of toxicology, identification is a key challenge and represents a bottleneck for a screening procedure. Furthermore, this task is much more challenging if a single analysis is possible due to the matrices’ availability, as is the case of screening using VAMS, which has to deal with a one-shot analysis. In this context, to ensure a reliable toxicological screening using the LC-HR/MS-Mitra^TM^ devices combination, the proper extraction of molecules from the tips is a fundamental step of the pre-analytical process. To optimize this step, we investigated different solvent mixtures and conditions to efficiently extract 90 molecules, selected as the most commonly detected drugs in clinical toxicological screening. In the literature, methanol is frequently reported to extract molecules from various matrices collected from Mitra^TM^ devices [25,26,27,28,29,30,31,32,33,34]. In some cases, an aqueous solution or buffer alone was used but supplemented with solid-liquid extraction [42], microextraction [35] or online extraction [39]. Our study highlighted that the use of an aqueous solution alone or a mixture of organic solvents alone does not lead to the highest extraction yields. A good compromise was obtained using a mixture of 30% organic phase and 70% water containing 1% formic acid. This mixture was adapted from a publication by Mandrioni et al. [33] in which we replaced 1% phosphoric acid with 1% formic acid. The use of an aqueous solution without any organic component allows the extraction of water-soluble compounds, such as metformin or baclofen, but is not optimal for extracting highly lipophilic molecules such as irbesartan. For these molecules, the use of an organic phase alone for extraction should, theoretically, result in better extraction yields compared to our mixture. However, the optimization step of our work has demonstrated that this is not really the case. One of the reasons could be related to the precipitation of hemoglobin by the solvents, which causes sequestration of the molecules within the precipitate; thus reducing extraction yield. On the other hand, it has already been reported that the addition of an aqueous component to the solvents can facilitate the extraction of some types of molecules [45], strongly supporting the results of this work.

Many publications use a combination of agitation and sonication steps to extract molecules from micro samplings. We also used these sample preparation steps, with the times optimized at 30 min for sonication and agitation. These times are much longer than those reported in the literature, in the order of a few minutes for the two steps [33,35,39,41,42]. It is well known that the optimization of extraction conditions for DBS or VAMS is complex and depends on the molecules to extract [46]. During a toxicological screening, the number of molecules to extract is potentially very high, with different chemical structures and physicochemical properties. As reported by Joye et al. [44], the diversity of the structures and properties of the molecules requires the use of the most versatile extraction process. The optimization of the extraction conditions is, therefore, a compromise, aimed at extracting molecules with the highest efficiency. In our conditions, 30 min of sonication and agitation were optimal to achieve the best yields for all the investigated molecules. It may, nevertheless, be regarded as a bottleneck point of the pre-analytical step, longer than those reported in the literature. Another limitation of our study includes the number of investigated molecules for the optimization of extraction conditions. Indeed, only ten molecules were assessed, but they were chosen adequately as they cover a broad array of the physicochemical properties of both licit and illicit drugs. Despite these limitations, the extraction yields obtained from the 90 compounds selected to establish the identification limits were all very satisfactory, with yields higher than 80% for all of the molecules identified in the 90 compounds tested. These yields are comparable to those reported in the literature [38,39,40,42]. Finally, in the clinical part of our study, the extraction yields calculated on the 119 identified compounds further confirm the excellent extraction yields, which are similar to those obtained from the 90 selected compounds.

Our extraction method and the analysis using LC-HR/MS enabled us to achieve identification limits lower than 12.5 ng·mL^−1^ for 82.2% of the 90 tested molecules. These limits are significantly higher than those reported by Mestad et al. [35] using Mitra^TM^ devices and by Joye et al. [44] using DBS. In these two publications, the detection limits were lower than 5 ng·mL^−1^ for the majority of the detected compounds. These values were established for the detection of drugs in cases of drivers under influence [35] or in a forensic context [44]. In the context of our study, our identification limits seem suitable for a reliable screening in clinical toxicology. Nevertheless, with our extraction process, some compounds, such as tetrahydrocannabinol (THC) and its metabolites, including THC-COOH, are not detected. These results are in line with those reported in the study performed by Mestad et al. [35]. On the opposite side, Joye et al. [44] demonstrated that it is possible to detect only THC-COOH from DBS after a double extraction process, including liquid extraction with a non-polar solvent. The lack of detection in our study cannot be attributed to the analytic method. In our study and in the study by Joye et al. [44], a screening using LC-HR/MS was developed, while LC-MS/MS was used in the publication of Mestad et al. [35]. As reported by Joye et al. [44], the percentage of THC-COOH detection in the samples varies depending on the type of support used to carry out the DBS, due to varying matrix effects based on the type of support. The absence of THC-COOH detection in our study may, thus, be due to poor extraction yield from the Mitra^TM^ device. Although reported in some publications [35], the lack of detection of cannabinoids and their derivatives remains a limiting point of our new screening method that needs to be optimized. The optimization of the pre-analytical step to extract these compounds from the Mitra^TM^ device remains a key point. The mixture extraction that we used contains a large part of water, which is not optimized for the extraction of very lipophilic compounds such as cannabinoid derivatives. In the near future, we will, thus, consider a two-step extraction to collect both hydrophilic and hydrophobic compounds; thus, allowing us to cover the broad array of molecular diversity responsible for intoxication, as recommended by Joye et al. [44]. This new pre-analytical approach should improve this lack of detection of cannabinoids.

A clinical application allowed us to demonstrate the feasibility of our new screening method. Fingertip sampling on the Mitra^TM^ device was performed by the nursing team upon admission of the patient, without the involvement of the laboratory team. The Mitra^TM^ devices were stored at room temperature on a special rack and extracted between 6 and 72 h after sampling. The stability of 90 molecules was evaluated over 72 h and no significant variation was observed. Our results are consistent with those of several other publications indicating excellent stability of the molecules, even the most fragile ones, such as cocaine, zopiclone and cathinones, on DBS [26,34,42,44] and on Mitra^TM^ [26,33,34,35,44]. The majority of the molecules are stable for one week at room temperature but can remain stable for several months when stored at −20 °C in alternative matrices, as previously reported [29].

The mass precision provided using LC-HR/MS in full-scan data-dependent MS2 (ddMS2) mode enables a more accurate identification of molecules during the screening procedure performed both on classical matrices [17,47,48] or alternative matrices such as DBS [42,44,49,50,51]. In routine laboratory use within a clinical toxicology context, this detection method is applied to plasma screening after the precipitation of proteins with acetonitrile containing various internal standards. To perform the screening on Mitra^TM^, we transposed our routine screening method to whole blood. In the 15 patients included in our study, we compared the number of molecules detected among three samples: plasma, whole blood and Mitra^TM^. From 20 µL of each of these samples, we obtained complete concordance in the detection between plasma and whole blood. The percentage of recovery was 98% between the whole blood and Mitra^TM^. This result confirms the finding of Protti et al. [26], which showed an excellent overlap in the number of compounds detected between plasma and VAMS or between plasma and DBS. In one patient, no drugs were detected suggesting the absence of false positives and the specificity of our method. In the remaining patients, we identified between 1 and 15 compounds from various therapeutic classes, with different structures and physical properties, including a natural compound, namely ricinine. The diversity of the compounds detected in our study is similar to that reported by Joye et al. [44] using DBS. In two patients, diazepam was not confirmed according to our criteria because the signal intensity was below 10^6^. This poor detection of diazepam has been previously reported by Mestad et al. [35]. The authors attribute this to non-optimized extraction conditions for diazepam due to its physicochemical properties. In our study, we observed the same phenomenon in our two patients, despite using extraction conditions that were completely different from those of Mestad et al. [35]. Moreover, the good detection limit that we obtained for diazepam in overloaded solutions (2.5 ng·mL^−1^) leads us to, rather, consider another explanation for this poor detection. In this study, it must be emphasized that the same molecules are detected in peripheral blood and capillary blood collected on Mitra^TM^ devices, suggesting a similar distribution of molecules between the two sampling points, as reported in several studies [52,53]. However, a hypothesis of the different distribution of diazepam between capillary and peripheral blood may explain the low detection of diazepam on Mitra^TM^ devices. This hypothesis will have to be verified with complementary studies to investigate other different distributions concerning other compounds not tested in this study. Another limitation of our study concerns the clinical part. In order to limit the impact of this study on the management of patients during admission, it was only possible to collect one Mitra^TM^ device for each patient, preventing us from testing the reliability of our collection conditions. However, to the best of our knowledge, this study is the first to compare toxicological screening performed on usual matrices, i.e., a plasma sample, to whole blood collected using a Mitra^TM^ device in real-life hospital intoxication cases. Even if only a single Mitra^TM^ device could be collected for each patient, the reliability of our approach has been illustrated thanks to the number of investigated patients, i.e., 15 cases.

## 4. Materials and Methods

### 4.1. Reagents

All investigated molecules (Supplementary Table S1) at the concentration of 1 mg·mL^−1^ in methanol, were purchased from Sigma-Aldrich™ (Saint-Quentin-Fallavier, France) and from LGC Group (Molsheim, France). Alprazolam-d5, amphetamine-d5, buprenorphine-d4, morphine-d3 and THC-COOH-d5 at the concentration of 0.1 mg·mL^−1^ in methanol were obtained from LGC Group (Molsheim, France). Ultrapure water, acetonitrile, methanol, formic acid (FA), and ammonium formate, all with a purity greater than 99% suitable for high resolution, were obtained from Fischer Chemicals™ (Illkirch, France). The whole blood, free of any targeted analytes, used for the preparation of the reference and working solutions was donated by the Etablissement Français du Sang of the CHU St Louis—Lariboisière—F. Widal.

### 4.2. Analytical Instruments

Toxicological screenings were achieved using ultra-high pressure liquid chromatography (LC, Vanquish™, Thermo Scientific, Illkirch, France) coupled with High-Resolution Q Exactive Focus™ mass spectrometry (Thermo Scientific, Illkirch, France) driven by Thermo™ Q Exactive Focus software (Version 4.1). LC was achieved on an Phenyl Hexyl column (Accucore®, 100 × 2.1 mm, 2.6 μm) (ThermoFisher Scientific, Bremen, Germany) maintained at 40 °C. Flow rate was set at 0.5 mL/min. The elution was based on a binary gradient system. Solvent A consisted of water with 2 mmol·L^−1^ ammonium formate and solvent B a methanol/acetonitrile mixture with 2 mmol ammonium formate (50:50). Both solvents A and B contained 0.1% formic acid. Eluent was maintained at 1% B for 1 min, increased to 99% B for 10 min, held at 99% B for 1.5 min before returning to 1% B, and finally, held for 4 min. The ionization voltage was set at 3.5 kV for positive ion mode and 2.5 kV for negative ion mode; sheath gas and auxiliary gas were 35 and 15 arbitrary units, respectively. S-lens RF was set at level 60; and vaporizer temperature and capillary temperature were both set at 320 °C. Nitrogen was used for spray stabilization, collision-induced dissociation experiments in the HCD cell, and as the damping gas in the C-trap. AGC target was fixed to 1 × 10^5^ for MS/MS experiments. Transient time was fixed to 120 and 50 milliseconds for full MS and MS/MS scans, respectively. Data were acquired in full-scan data-dependent MS2 (dd-MS2) mode. In this mode, a full-scan acquisition was performed at a resolution of 70,000 between *m*/*z* 100 and 1000, followed by the acquisition of MS/MS spectra of the three most intense ions. The following settings for the dd-MS2 mode were used: resolution of 17,500, isolation window of 1.0 and higher-energy collisional dissociation (HCD) cell with stepped normalized collision energy of 17.5, 35.0, and 52.5 V.

### 4.3. Preparation of the Solutions

*Stock solution*. A stock solution containing 90 compounds, parent molecules and metabolites (see: Supplementary Table S1) at the concentration of 500 ng·mL^−1^, was prepared by spiking 5 mL of blank whole blood with different dilutions of all compounds in a water/methanol mixture (50/50%, *v*/*v*). This stock solution was stable for 1 month at +4 °C.

*Working solutions.* The stock solution was diluted in «free» whole blood to obtain 9 working solutions containing the 90 compounds at final concentrations of 250, 125, 100, 50, 25, 12.5, 5.0, 2.5 and 1.25 ng·mL^−1^, respectively. These solutions, prepared under a final volume of 1 mL, were stable for 1 month at +4 °C.

*Extraction mixtures.* Five extraction mixtures containing varying proportions of three solvents, water with 1% (*v*/*v*) formic acid, methanol and acetonitrile, were tested (Table 1).

*Internal standards solutions*. A stock solution containing alprazolam-d5 amphetamine-d5, buprenorphine-d4, morphine-d3 and THC-COOH-d5, was prepared by diluting 20 µL of each 0.1 mg·mL^−1^ solution in methanol. This solution was stable for 6 months at −20 °C. A working solution was prepared by diluting the stock solution in 1:1000 methanol to obtain a final concentration of 30 ng·mL^−1^, for each internal standard. This solution is stable for 2 weeks at +4 °C.

*Recovery solution.* The recovery solution was a mixture (80/20%, *v*/*v*) of water/acetonitrile with 1% (*v*/*v*) FA.

### 4.4. Process to Fill Volumetric Absorptive Microsamplings

Volumetric absorptive microsampling (VAMS) devices were purchased from Trajan Scientific and Medical (Milton Keynes, UK) under the brand name Mitra^TM^. Twenty microliter (20 µL) Mitra^TM^ devices were chosen to realize all the experimental protocols. They were filled with whole blood by lightly touching the blood’s surface with the tip for 5 s, without submerging it. An additional 2 s after the tip became fully red was added, as recommended by the manufacturer, to ensure complete absorption of the 20 µL sample. The Mitra^TM^ devices were then left to dry at room temperature in the dark for at least 6 h, as advised by the supplier, without any prior extraction. This process was applied to fill the Mitra^TM^ devices with either whole blood drops or from a heparinized tube.

### 4.5. Experimental Protocols

*Step 1: Optimization of the extraction conditions from Mitra^TM^ devices.*

We selected 10 molecules (amphetamine, baclofen, cocaine, cyamemazine, diazepam, diltiazem, irbesartan, propranolol, morphine and tramadol) to be included in the working solutions. These compounds have different structures and solubilities and were chosen to represent the molecules commonly found in toxicological screenings. Two working solutions, at concentrations of 250 ng·mL^−1^ and 25 ng·mL^−1^, were investigated using various extraction mixtures (Table 1), with varying sonication and agitation times, as well as different time delays between filling the Mitra^TM^ devices and the extraction process (6, 12, 24 and 48 h). For each solution and each parameter, the experiments were performed in triplicate. The intensity of the response was evaluated using the chromatographic peak areas for each compound.

*Step 2: Determination of identification limits and extraction yields for 90 compounds sampled using Mitra^TM^ devices.*

To determine the identification limits, three Mitra^TM^ devices were filled with 9 working solutions containing 90 molecules tested at concentrations ranging from 1.25 to 250 ng·mL^−1^ according to the protocol previously described. The Mitra^TM^ devices were then placed on a special rack and stored under the conditions previously mentioned. The Mitra^TM^ devices were extracted under the optimized extraction conditions established in step 1.

To determine the extraction yields, the chromatographic areas obtained from one Mitra^TM^ device and 20 µL of whole blood for each working solution were compared. Both the Mitra^TM^ devices and the 20 µL of whole blood were extracted under the optimized extraction conditions established in step 1.

*Step 3: Clinical application.*

The clinical study was conducted according to the Helsinki principles, declared to the Commission Nationale de l’Informatique et des Libertés (declaration number, 2067659), and approved by the ethics committee of the French Society of Intensive Care (protocol number, FICS20020231). Due to the minimal interventional design of the study, the requirement for written consent was waived. Between March and April 2022, our new toxicological screening approach using Mitra^TM^ devices was tested on fifteen patients admitted to the Toxicological Critical Care Unit of the University Hospital, who were suspected or confirmed to have undergone voluntary poisonings. Upon admission, all patients were simultaneously sampled using a Mitra^TM^ device by lightly touching the surface of a whole blood drop at the fingertip, and also at the elbow to obtain a conventional heparinized sample. All samples were collected by the nursing team without the involvement of laboratory staff. The Mitra^TM^ devices were then placed on a special rack. The rack and the heparinized samples were kept at room temperature in the clinical unit and collected by the laboratory team between 6 and 48 h after sampling. After collection, 20 µL of whole blood and 20 µL of plasma per patient were introduced into Eppendorf^TM^ tubes. The Eppendorf^TM^ tubes and the Mitra^TM^ devices were extracted under previously described conditions. To limit the impact of this study on the management of patients admitted to the toxicological critical care unit, each patient was sampled only once on a Mitra^TM^ device.

### 4.6. Extraction Process

Before extraction, the tips of the Mitra^TM^ devices were cut off and placed in Eppendorf^TM^ tubes. In each Eppendorf^TM^ tube containing 20 µL of the sample (plasma, whole blood, and tip), 500 µL of the extraction solution (75% water with 1% FA, 10% methanol, 15% acetonitrile) and 20 µL of internal standards solution were added. The extraction process was performed using sonication for 30 min followed by 30 min of agitation at room temperature. The supernatants were recovered and evaporated to dryness under a nitrogen stream at 40 °C. The dry residues were redissolved in 50 µL of the recovery solution, and then 10 µL were injected into the chromatographic system.

### 4.7. Data Analysis

The MS and fragmentation data acquired were processed using the Thermo Scientific Forensic TraceFinder™ software version 4.1. This specific software performs a thorough interrogation of the database by utilizing its built-in database and the Lariboisière mass spectral library of 1560 compounds. Different parameters, such as retention times, fragment and isotope patterns, and library matching were used to detect and confirm the drugs and metabolites. In our study, the detection of compounds was assessed by a signal intensity above 1.10^6^ and confirmed by total concordance between the experimental and library retention times, complete fragments and isotopic patterns, and by cross-matching the values above 70%. The detection and confirmation of the 5 internal standards in each sample allowed us to validate the pre-analytical and analytical phases of our method.

A complete validation was not carried out. Only the identification limits were determined as previously reported [54,55]. To establish these identification limits, we selected 90 molecules among the most frequently encountered in our daily toxicological screening activity. As reported in Table S1, these molecules have chemical structures and log *p* values that are representative of legal and illegal drugs that can be detected in our LC-HR/MS toxicological screening process. The identification limits of each molecule were established as the smallest concentration that allowed us to obtain a chromatographic area higher than 10^6^ with a signal/noise ratio higher than 3, and total concordances between the experimental and library retention times, complete fragments and isotopic patterns, and cross-matching values above 70%.

In Section 1, for the optimization of the method, the results are expressed as the mean ± standard deviation with *n* = 3.

## 5. Conclusions

Our work demonstrates the possibility of performing a reliable toxicological screening from 20 µL of whole blood by coupling HR/MS with microsampling using Mitra^TM^ devices. The feasibility of our method was validated by a clinical study in patients hospitalized for intoxication. Our original screening approach opens new insights in numerous toxicologic fields, such as (*i*) pediatrics, for small volume sampling; (*ii*) forensics, for small volume sampling and blood stain analysis; (*iii*) massive toxicological screening during events; or (*iv*) facilitating inter-laboratory post-exchange. Other possibilities that can be suggested are addictology or epidemiology studies realized using Mitra^TM^ microsampling. A new goal will be to combine reliable quantification using Mitra^TM^ devices and toxicological screening, to confirm and complete the interest in microsampling in clinical toxicology.

## Figures and Tables

**Table 1:** Composition of different extraction mixtures containing various proportions of 3 solvents: water + formic acid (FA) 1% (*v*/*v*), methanol and acetonitrile.

Mixture	1	2	3	4	5
Water, 1% FA (%, *v*/*v*)	100	75	50	25	0
Methanol (%, *v*/*v*)	0	10	25	37.5	50
Acetonitrile (%, *v*/*v*)	0	15	25	37.5	50

## Data Availability

Not applicable.

## Supplementary Materials

The following supporting information can be downloaded at: https://www.mdpi.com/article/10.3390/molecules28083466/s1, Table S1: List, in alphabetical order, of the 90 compounds, parent molecules and metabolites, with log *p* values included in this study.

## Sample Availability

Samples of the compounds are not available from the authors.
