# Supplementary material for: New Trend in Toxicological Screening Using Volumetric Absorptive Microsampling (VAMS) and High-Resolution Mass Spectrometry (HR/MS) Combination
Source: Molecules. 2023 Apr 14;28(8):3466. doi: 10.3390/molecules28083466 (PMC10141006; doi:10.3390/molecules28083466)
Supplement: Supplementary file 1 [file molecules-28-03466-s001.zip › molecules-2259219-supplementary.pdf]

Supplementary Materials

New Trend in Toxicological Screening Using Volumetric Absorptive Microsampling (VAMS) and High-Resolution Mass Spectrometry (HR/MS) Combination

Pascal Houzé[1,2*], Ilona Borowski[1], Eugénie Bito[1], Romain Magny[1,3], Athina Morcos[1], Sebastian Voicu[3,4], Bruno Mégarbarne[3,4] and Laurence Labat[1,4]

[1]Laboratory of Toxicology, Federation of Toxicology, Lariboisière Hospital, Assistance Publique-Hôpitaux de Paris (AP-HP), 10 rue Ambroise Paré, 75010 Paris, France

[2]Chemical and Biological Health Technologies Unit (UTCBS), CNRS UMR8258-U1022, University of Paris, 4 Avenue de l'Observatoire, 75006 Paris, France

[3]INSERM UMRS-1144, University of Paris, 4 Avenue de l'Observatoire, 75006 Paris, France

[4]Department of Medical and Toxicological Critical Care, Federation of Toxicology, Lariboisière Hospital, Assistance Publique-Hôpitaux de Paris (AP-HP), 10 rue Ambroise Paré, 75010 Paris, France

*Correspondence: pascal.houze@aphp.fr; Tel.: +33-1-44-49-65-86

Supplementary Table S1: List, in alphabetical order, of the 90 compounds, parent molecules and metabolites, with log P values, included in this study. 6-MAM : 6-monoacetylmorphine; 11-hydroxy-THC: 11-hydroxy-Δ9-tetrahydrocannabinol; LSD: lysergic acid diethylamide; MBDB: N-methyl-1-(1,3-benzodioxol-5-yl)-2-aminobutane; MDA: 3,4-méthylènedioxyamphétamine ; MDEA: 3,4-méthylènedioxy-N-éthylamphétamine ; MDMA: 3,4-methylenedioxy-N-methylamphetamine; MDPV: Methylenedioxypyrovalerone; THC : tetrahydrocannabinol; THC-COOH : 11-carboxy-tetrahydrocannabinol. * log P values were obtained from Drugs Bank data base

Number	Name	log P value*	Number	Name	log P value*
1	3-chloromethcathinone	1.86	46	MBDB	2.15
2	4- methylmethcathinone	2.39	47	MDA	1.56
3	6-MAM	1.31	48	MDEA	- 0.72
4	7-amino-clonazepam	2.38	49	MDMA	2.15
5	11-hydroxy-THC	5.33	50	MDPV	3.97
6	acebutolol	1.71	51	metamphetamine	2.07
7	acetylcodeine	2.00	52	methadone	3.93
8	alimemazine	4.71	53	methylester ecgonine	0.14
9	alprazolam	2.12	54	methylone	1.91
10	amphetamine	1.76	55	methylphenidate	0.2
11	aripiprazole	5.3	56	midazolam	4.33
12	baclofen	1.3	57	morphine	0.87
13	benzoylecgonine	- 0.59	58	N-acetyldiltiazem	2.73
14	bromazepam	2.05	59	N-desmethyltramadol	2.07
15	buprenorphine	4.98	60	N-desmethylvenlafaxine	2.36
16	clonazepam	2.41	61	nitrazepam	2.25
17	clozapine	3.23	62	norbenzoylecgonine	- 0.8
18	cocaethylene	2.64	63	norbuprenorphine	2.3
19	cocaine	2.30	64	norcodeine	0.96
20	codeine	1.39	65	nordazepam	3.21
21	cyamemazine	4.27	66	norfentanyl	1.42
22	dehydroaripiprazole	4.98	67	norfluoxetine	3.74
23	desmethylclozapine	3.02	68	noroxycodone	0.65
24	desmethylzopiclone	- 0.09	69	O-desmethyltramadol	1.72
25	dextromethorphan	3.97	70	O-desmethylvenlafaxine	2.72
26	diazepam	2.82	71	oxazepam	2.24
27	dihydrocodeine	0.8	72	oxycodone	0.7
28	diltiazem	2.7	73	phenacetin	1.58
29	diphenhydramine	3.27	74	phenobarbital	1.47
30	ecgonine	- 1.8	75	pregabalin	1.78
31	ethylglucuronide	- 1.6	76	propanolol	3.48
32	fentanyl	4.05	77	propofol	3.79
33	fluoxetine	4.05	78	secobarbital	1.97
34	furosemide	2.03	79	sufentanil	3.95
35	gabapentin	1.25	80	temazepam	2.19
36	haloperidol	4.3	81	tetrazepam	3.20
37	hydrochlorothiazide	- 0.07	82	THC	5.65
38	hydromorphone	1.06	83	THC-COOH	5.14
39	irbesartan	4.5	84	tramadol	1.34
40	ketamine	3.12	85	triazolam	2.42
41	lamotrigine	1.93	86	tropatepine	4.82
42	levamisole	2.3	87	valsartan	1.50
43	lorazepam	3.35	88	zaleplon	0.9
44	lormetazepam	3.39	89	zolpidem	3.02
45	LSD	2.95	90	zopiclone	0.8
